# Additively Manufactured Bionic Corrugated Lightweight Honeycomb Structures with Controlled Deformation Load-Bearing Properties

**DOI:** 10.3390/ma17102274

**Published:** 2024-05-11

**Authors:** Jie Li, Han Wang, Xianghao Kong, Zhiwei Jiao, Weimin Yang

**Affiliations:** 1 College of Mechanical and Electrical Engineering, Beijing University of Chemical Technology, 15 East North Third Ring Road, Beijing 100029, China; 2 China Academy of Safety Science and Technology, Security Building, Building A 1, 32 Beiyuan Road, Chaoyang District, Beijing 100012, China; wnghan0414@163.com

**Keywords:** bionic corrugated construction, additive manufacturing, controlled deformation, load-bearing

## Abstract

The rapid development of additive manufacturing (AM) has facilitated the creation of bionic lightweight, energy-absorbing structures, enabling the implementation of more sophisticated internal structural designs. For protective structures, the utilization of artificially controlled deformation patterns can effectively reduce uncertainties arising from random structural damage and enhance deformation stability. This paper proposed a bionic corrugated lightweight honeycomb structure with controllable deformation. The force on the onset state of deformation of the overall structure was investigated, and the possibility of controlled deformation in the homogeneous structure was compared with that in the corrugated structure. The corrugated structures exhibited a second load-bearing capacity wave peak, with the load-bearing capacity reaching 60.7% to 117.29% of the first load-bearing peak. The damage morphology of the corrugated structure still maintained relative integrity. In terms of energy absorption capacity, the corrugated lightweight structure has a much stronger energy absorption capacity than the homogeneous structure due to the second peak of the load carrying capacity. The findings of this study suggested that the combination of geometric customization and longitudinal corrugation through additive manufacturing offers a promising approach for the development of high-performance energy-absorbing structures.

## 1. Introduction

The rapid development of additive manufacturing (AM) has facilitated the development of lightweight, energy-absorbing structures that enable more sophisticated internal structural designs. Destructive impact behavior occurs in many scenarios, often resulting in irreversible and serious hazards with severe consequences for equipment and even personnel lives and is mostly unpredictable. For protective structures, artificially controlled deformation patterns can effectively reduce the uncertainty generated by random structural damage and enhance deformation stability. In aerospace, the automotive industry, biomedical engineering, building interiors, etc., lightweight, high-strength mechanical structures with energy-absorbing features show higher crashworthiness, lower fuel consumption, and improved safety of people’s lives.

Humans have a long history of exploration in the field of bionics. Nature’s natural bionic structures have proven to be an efficient and superior solution through billions of years of evolution and natural selection [1]. Humans have continued on the journey of bionics, analyzing, dissecting, learning, and absorbing the potential of natural materials in nature, and have been inspired to develop a range of new structures and materials ideal for energy absorption. One of the most successful was a honeycomb material designed to mimic a honeycomb [2,3]. Researchers continue to explore the properties of bionic honeycomb in terms of structure, energy absorption, blast resistance, and impact resistance [4]. In addition, honeycomb materials with even better performance have been developed by improving and upgrading the natural materials. Inspired by bamboo, Hu et al. [5]. investigated the axial extrusion energy absorption properties of tubular nested structures. Combining the bionic structure with the tubular structure enhances the energy absorption characteristics. Liu et al. [6] introduced the design concept of durian shell into the helmet liner and designed a bi-directionally staggered rectangular flat truncated helmet liner, which significantly improves the energy absorption characteristics. Zhou et al. [7] proposed a bionic sandwich structure based on the seagull feather, which optimizes and improves the traditional honeycomb. In addition, inspired by the layered structure of biomaterials, Li et al. developed a series of layered honeycomb structures [8]. The upgrading and improvement of the traditional honeycomb is the next effective means to continuously explore the excellent performance.

Lightweight structures represented by cellular structures have excellent performance in terms of specific stiffness, specific strength, specific absorption capacity, buckling resistance, and toughness. This cellular structure can absorb a large amount of energy during deformation and buckling collapse, and the irreversible deformation that occurs locally effectively reduces the destructive impact of the entire structure or protects important internal components (e.g., protective helmets, landing gear for astronauts) [9,10,11]. Meanwhile, honeycomb, as a representative of lightweight structures, is widely used as the core of sandwich structures [12]. The honeycomb form, as a kind of porous structure, has a diversity of microstructures, and Wang et al. designed a random honeycomb cylindrical shell structure constructed in a procedural manner to study and evaluate the deformation patterns and mechanical properties [13]. Drawing on the functional gradient fabrication method and personalized design of lattice structures [14], the mechanical and physical compatibility of honeycomb structures can be maximized. In addition, the well-designed structure has a stronger energy absorption capacity [15,16], and the design method of constructing solid geometries is optimized through topological optimization and gradient design [17].

The rapid development of additive manufacturing technology in recent years has provided great potential for structural applications [18]. Due to the direct output-oriented nature of additive manufacturing, the variability and flexibility of structural design can be significantly enhanced [19]. Ning et al. introduced gradient nanostructures through additively fabricated alloys, which enhanced the heterogeneous deformation-induced hardening effect and twinning/microstrip behavior activated in the matrix region at 88 K [20]. Based on Zhang et al.’s description, additive manufacturing incorporates multifunctional structural design such as load-bearing, electronics, thermal conductivity, and radiation protection [21], realizing structure–function integration. In addition, lattice structures fabricated by additive manufacturing are outstanding in energy absorption [9,15,16,22], and additive manufacturing technology plays an important role in the fabrication of complex structures and biomimetic biomaterial components [14,23].

Energy absorption is a passive protection method aimed at converting energy from one form to another during impact, thus reducing damage to core components [24]. Targeted energy absorption through controlled deformation design is a powerful means to enhance structural reliability. Controlled deformation design is a more efficient and adaptive optimization method. Through the active design of geometrical and physical parameters, it achieves a stable and predictable deformation pattern of the structure to produce a reliable mechanical response [22,24,25]. The desired shape deformation or performance change is achieved through a series of structural optimization design methods, such as topology optimization, shape optimization, and size optimization [24]. Yang et al. integrated the controllable deformation design with the shape memory function and prepared a smart-responsive bilayered hydrogel, which exhibited controllable deformation recovery performance and had excellent anti-fatigue properties [26]. Currently, most of the research on controlled deformation focuses on shape memory materials, mechanical metamaterials, and lattice structures [15,16,26,27,28]. Some studies focus on structural properties, exploring the effects of material properties such as strength and modulus and geometrical parameters such as honeycomb wall thickness and height, and core shape, on the energy-absorbing characteristics [29,30,31,32,33,34,35,36]. Several authors [37,38,39] studied the impact response of sandwich cores with different density gradient distributions and noted that gradient cores are superior to uniform cores in terms of energy absorption. By introducing gradient properties, better energy absorption is achieved with progressive or multilayer cell structures [40]. Controlled deformation of novel lightweight honeycomb structures has not been widely studied.

Currently, researchers have focused on the two-dimensional aspects of cellular uniform wall thickness, geometry, rigid instability state, energy absorption characteristics, and two-dimensional topology optimization [22,25]. The impact response of geometrically tailored honeycomb structures with linearly varying cell wall thickness in the thickness direction [9,41] has been studied. There are some studies performed on reinforced corrugated sandwich panels. MRM Rejab [42] has fabricated sandwich panels with homogeneous corrugated cores by using hot press moulding technique, showing more advantages in compression performance. A.K. Haldar [43] has studied the homogeneous triangular and trapezoidal cores in terms of compressive strength and energy absorption capacity. In this paper, an improvement is made on the basis of the three geometrical designs studied by Andrew et al. to propose a controllable deformation corrugated lightweight honeycomb, aiming to control the deformation onset point of the lightweight honeycomb in the artificially created range, so as to control the state of deformation onset and the fragmentation morphology; finite element software is used to simulate the gradient-constructed and homogeneous geometrically tailored lightweight honeycomb, respectively, in order to validate the designed controllable deformation onset point; the designed controllable deformation onset point is verified through quasi-static compression experiments, the compressive capacity of the three types of lightweight honeycombs is analyzed, and the energy absorption characteristics are studied comparatively.

## 2. Experimental Procedure

### 2.1. Geometrical Design and 3D Printing

**Figure 1:**
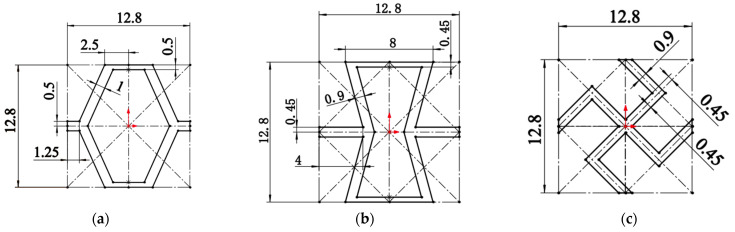
Sectional geometric design dimensions of the three structures: (**a**) irregular hexagons, (**b**) reconstructed hexagons, and (**c**) chiral structures (in mm). (All dimensions are in mm; The red arrow is the origin of the center of the coordinates).

First, the three-dimensional model is built. In order to investigate geometric clipping and ripple gradient effects, hexagonal honeycomb samples with 3 × 3 cells h = 40 mm were designed and manufactured. The samples in this study comprise three geometric configurations: irregular hexagon, reconfigurable hexagon, and chirality. Additionally, two longitudinal gradients were incorporated: a homogeneous gradient and a ripple gradient. These sample configurations are illustrated in Figure 1.

**Figure 2:**
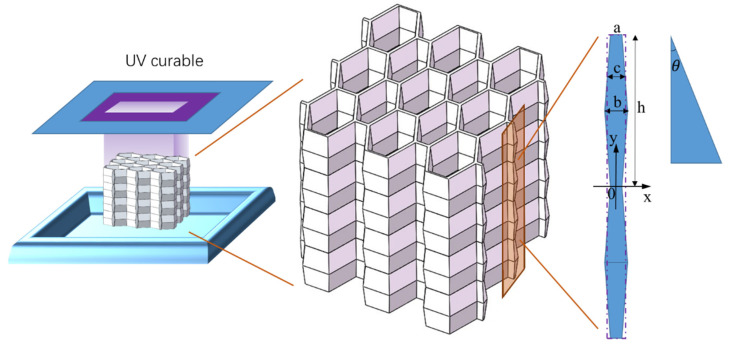
Corrugated lightweight honeycomb production process.

The honeycomb samples were fabricated using 3D printing technology, with continuous fusion and deposition of the material. The viscosity, reactivity, and curing process of the photopolymer were carefully controlled (physical parameters of the material are provided in Table 1). Subsequently, the samples were cleaned, cured, and any remaining resin residue was removed. To achieve honeycomb curing, the samples were exposed to ultraviolet light for a duration of 6 min, as depicted in Figure 2.

The geometrically customized honeycomb structures were created by non-linearly varying the cell wall thickness along the penetration thickness direction (y). In Figure 2, the values of *a*, *b*, and *h* were determined through design optimization to ensure that the three cellular structures have the same relative density. *c* is the equivalent average thickness. Variable thickness corrugation of the wall causes an inclination of the wall to the plumb line. The formula for the inclination of wall thickness is as follows:

(1) $$\theta =arcsin\frac{2h}{\sqrt{a^{2}+b^{2}+4h^{2}-2ab}}$$

where θ is the slope of the wall thickness, a is the short side of the ripple, *b* is the long side of the ripple, and *h* is the height of the single ripple.

The longitudinal section outline is

(2) $$f\left(x\right)=\left\{\begin{array}{ll} \frac{h}{b-a}x-\frac{ah}{2\left(b-a\right)} & \frac{a}{2}<x<\frac{b}{2},0<f\left(x\right)<\frac{h}{2}\\ -\frac{h}{b-a}x+\frac{2bh-ah}{2\left(b-a\right)} & \frac{a}{2}<x<\frac{b}{2},\frac{h}{2}<f\left(x\right)<h \end{array}\right.$$

**Figure 3:**
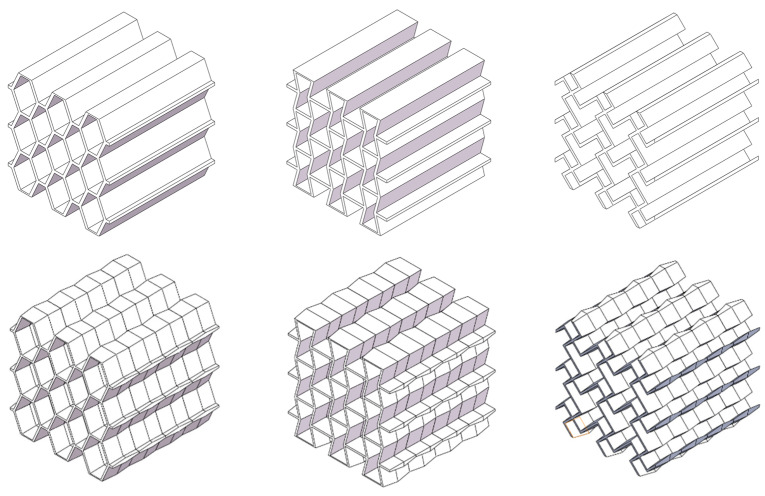
Six lightweight honeycomb models.

Three corrugated lightweight honeycomb models were constructed, as shown in Figure 3. The sample material was photosensitive resin C-UV 9400 (Dongguan Aide Synthetic Material Technology Co., Dongguan, Guangdong Province, China). The following table (Table 1) shows the physical and mechanical parameters of the C-UV 9400 resin.

The load capacity of the 40 mm × 40 mm × 40 mm model exceeded the maximum range of the equipment used for the experiment. Therefore, the model could not be completely crushed. Therefore, in order to obtain a complete force-displacement curve, we used a model cell with a separate honeycomb structure, i.e., 20 mm high (i.e., 1/18 of the sample).

### 2.2. Preliminary Stress Analysis in Quasi-Static Compression

**Figure 4:**
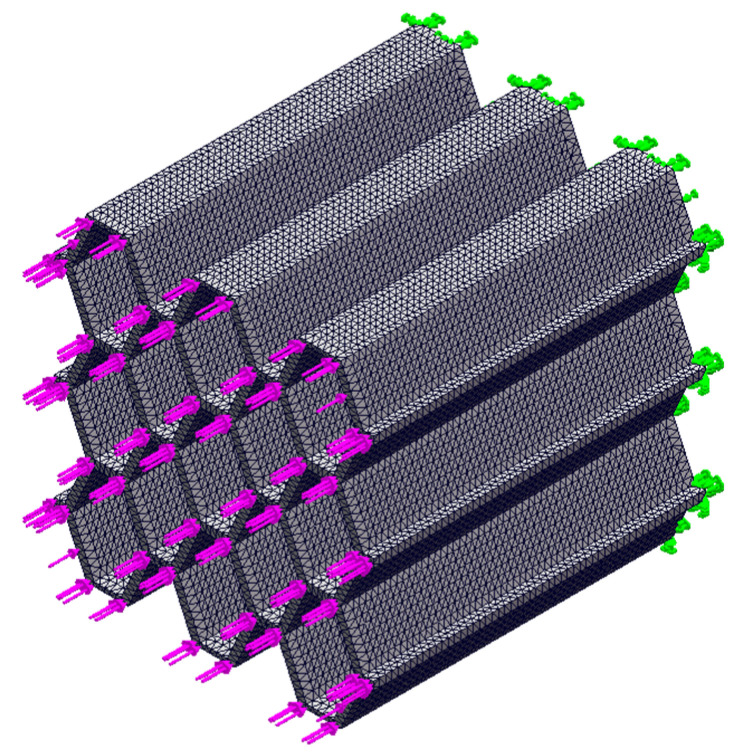
Schematic diagram of pre-processing for finite element simulation. (Green is constraint, red is load).

The quasi-static compression process was simulated using ANSYS 19.0 finite element software, with a focus on analyzing the structural force deformation during the initial stage of deformation. Finite element modeling was conducted on six lightweight honeycomb structures, including the homogeneous irregular hexagonal structure, homogeneous reconstructed hexagonal structure, homogeneous chiral structure, corrugated irregular hexagonal structure, corrugated reconstructed hexagonal structure, and corrugated chiral structure. A uniform load of 1000 N was applied to the longitudinal plane, while the bottom surface was fixed. The simulation assumed that the structure underwent compression without lateral displacement, the material was homogeneous, and the honeycomb sidewalls were free surfaces. The simulation setup was designed to mimic quasi-static compression experiments, as depicted in Figure 4.

To ensure accurate mesh applicability, a tetrahedral mesh (Solid 92) was employed. The material chosen for the simulation was the 9400 resin, which was the same as that used in the experimental study (refer to Table 1 for more details). The grid data for the delineation was determined based on the validation of grid independence and is provided in Table 2. The simulation focused on reproducing the initial force state when the structure experienced compression and analyzing the point of structural damage during the initial transient state.

The main reason why bionic lightweight structures have good cushioning performance is that they can exhibit a series of deformation processes under compressive loads [44]: linear elastic deformation–elastic buckling–plastic collapse–brittle damage. The location of the initial deformation of the material directly determines the overall deformation pattern of the structure. Therefore, finding the location of the maximum stress point of the model during the elastic–plastic deformation phase is an important part of predicting the controllable deformation of the structure. The simulated stresses during the elastic–plastic phase follow the Von Mises failure criterion in order to investigate the location of the onset of stress deformation in the structure.

The modulus $E_{3}^{*}$ during linear elastic deformation in the x_3_ direction reflects the modulus *E_s_* of the load-bearing cross section, and the modulus of elasticity of a uniform wall thickness hexagonal honeycomb is the following:

(3) $$E_{3}^{*}=\frac{E_{s}\rho^{*}}{\rho_{s}}=\left[\frac{d/(l+2)}{2(d/l+sin\theta )cos\theta }\right]\frac{t}{l}E_{s}$$

In elastic buckling in the x_3_ direction, the load is the sum of the borehole wall bearing and the collapse stress is the following:

(4) $${\left(\sigma_{el}^{*}\right)}_{3}\approx \frac{2}{1-v_{s}^{2}}\frac{\frac{l}{d+2}}{(d/l+sin\theta )cos\theta }{\left(\frac{t}{l}\right)}^{3}E_{s}$$

During plastic collapse in the x_3_ direction, the cross-section stress exceeds the material yield strength $\sigma_{ys}$, the pore wall asymptotically collapses, the folding wavelength is approximately equal to the pore edge length, and the plastic collapse stress is the following:

(5) $${\left(\sigma_{pl}^{*}\right)}_{3}\approx \frac{\pi }{4}\frac{\frac{d}{l+2}}{(d/l+sin\theta )cos\theta }{\left(\frac{t}{l}\right)}^{3}\sigma_{ys}$$

In the case of brittle damage in the x_3_ direction, the cross-section stress exceeds the material tensile strength $\sigma_{fs}$, which is the upper limit of the tensile strength of brittle materials:

(6) $${\left(\sigma_{f}^{*}\right)}_{3}=\frac{\rho^{*}}{\rho^{s}}\sigma_{fs}$$

### 2.3. Quasi-Static Compression Process and Energy Absorption Test

**Figure 5:**
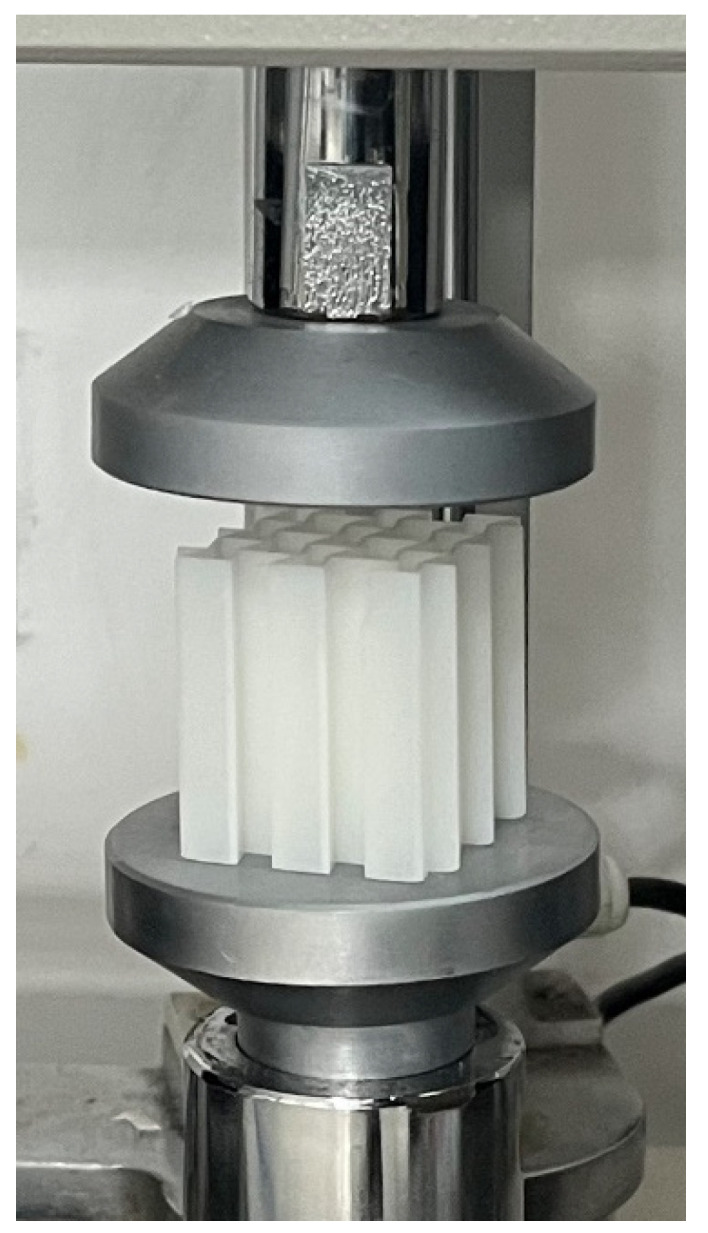
Testing process of the samples.

The quasi-static compression process of 3D printed samples was tested using a WDT-W micro-controlled electronic universal testing machine (Figure 5) with a loading rate of 0.1 mm/s to determine the force and load-bearing properties of the designed corrugated lightweight honeycomb structure and the homogeneous honeycomb structure of the comparison samples. The important quasi-static compression process per transient load carrying capacity variation curves were obtained. In addition, the energy absorption curves were obtained by integral calculation.

The evaluation of the energy absorption capacity is a key indicator of the mechanical properties, and the integration of the force–displacement curve with the stress–strain curve is the way to express the compression process:

(7) $$EA=\int_{0}^{\varepsilon_{t}}\sigma d\varepsilon$$

(8) $$Wc=\int_{0}^{t}FdT$$

## 3. Results and Discussion

### 3.1. Preliminary Stress Analysis

**Figure 6:**
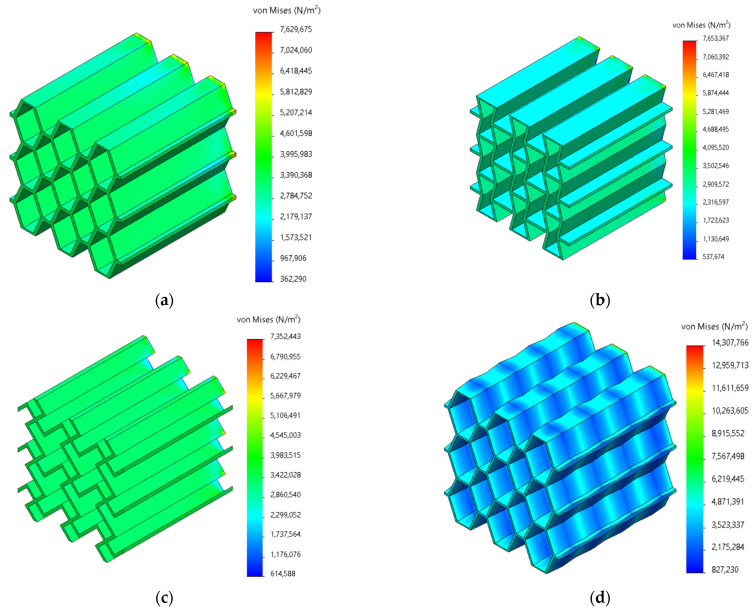

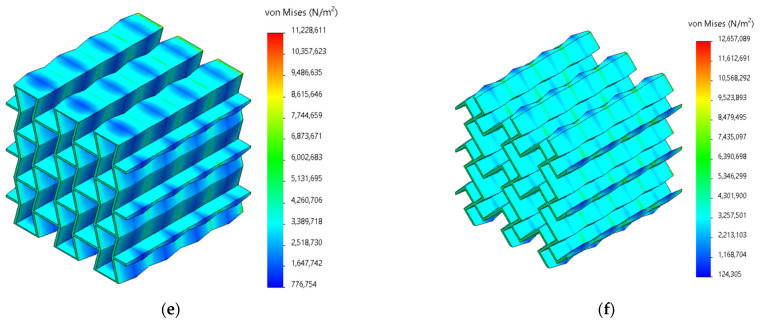
Light honeycomb stress simulation of the (**a**) homogeneous irregular hexagonal structure, (**b**) homogeneous reconstructed hexagonal structure, (**c**) homogeneous chiral structure, (**d**) corrugated irregular hexagonal structure, (**e**) corrugated reconstructed hexagonal structure, and (**f**) corrugated chiral structure.

In this section, we simulated the quasi-static compression characteristics of a variety of structures. The focus is on the forces during the elastic–plastic deformation phase at the beginning of the deformation of the structure. According to the stress contour of different structures under the same force, it can be seen that the overall stress distribution of homogeneous structures is uniform (as shown in Figure 6a–c), and it was almost impossible to predict the location and mode of failure in advance, as well as that the deformation was in an uncontrollable situation. On the other hand, the homogeneous structure is more uniformly stressed as a whole, which is not conducive to the purpose of preferential deformation at the intended location. A comparative study of the corrugated structure (Figure 6d–f) showed that the stress ripple has regular fluctuations. After the positive pressure was applied, the narrow edge of the designed ripple was preferentially deformed to achieve the purpose of controllable deformation. The stress concentration phenomenon of the support end of the corrugated structure was improved, the possibility of local failure was lower, and the overall stability of the structure was higher.

**Figure 7:**
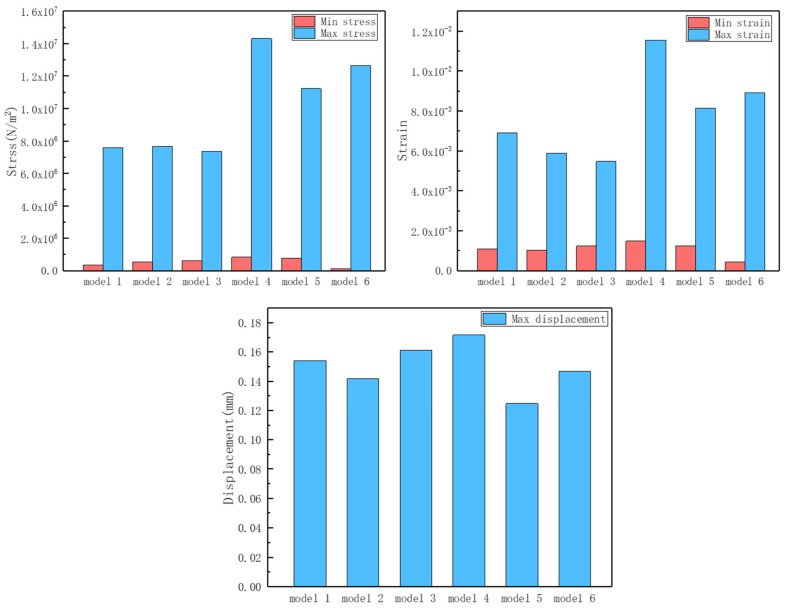
Max/min mechanical properties for 6 models.

According to the maximum/minimum stresses, maximum/minimum strains, and maximum displacements (Figure 7) of the six models (see Table 2), the controllable deformation of the corrugated structure is feasible. The difference between the minimum stress and minimum strain of the six models is relatively small. The maximum stress and maximum strain of the three corrugated structures increased significantly. Combined with the contour diagrams, it can be seen that the maximum stress–strain occurs at the “narrow side” of the designed corrugations. Therefore, the occurrence of deformation can be sensed in advance at the maximum stress–strain corrugation. This is conducive to the controllable deformation design of the overall structure and the regular deformation during the elastic deformation stage, so that the subsequent brittle damage of the overall structure is no longer “disordered”. The pre-sensing of destructive brittle deformation can be realized at the early stage of structural design. At the same time, the corrugated structure enhances the “elasticity” to a certain extent, delaying the occurrence of brittle damage and changing the performance of the structure at the initial stage of deformation.

### 3.2. Transient Bearing Capacity Change Curve and Failure Morphology

In order to reflect the deformation law of the corrugated honeycomb lightweight structure more directly and effectively, the experimental steps in 2.3 were adopted to verify the single cell sample with h = 20 mm. The transient bearing capacity curves of the three designed lightweight structures were obtained, and the failure morphology of the lightweight structures was observed after the experiment.

**Figure 8:**
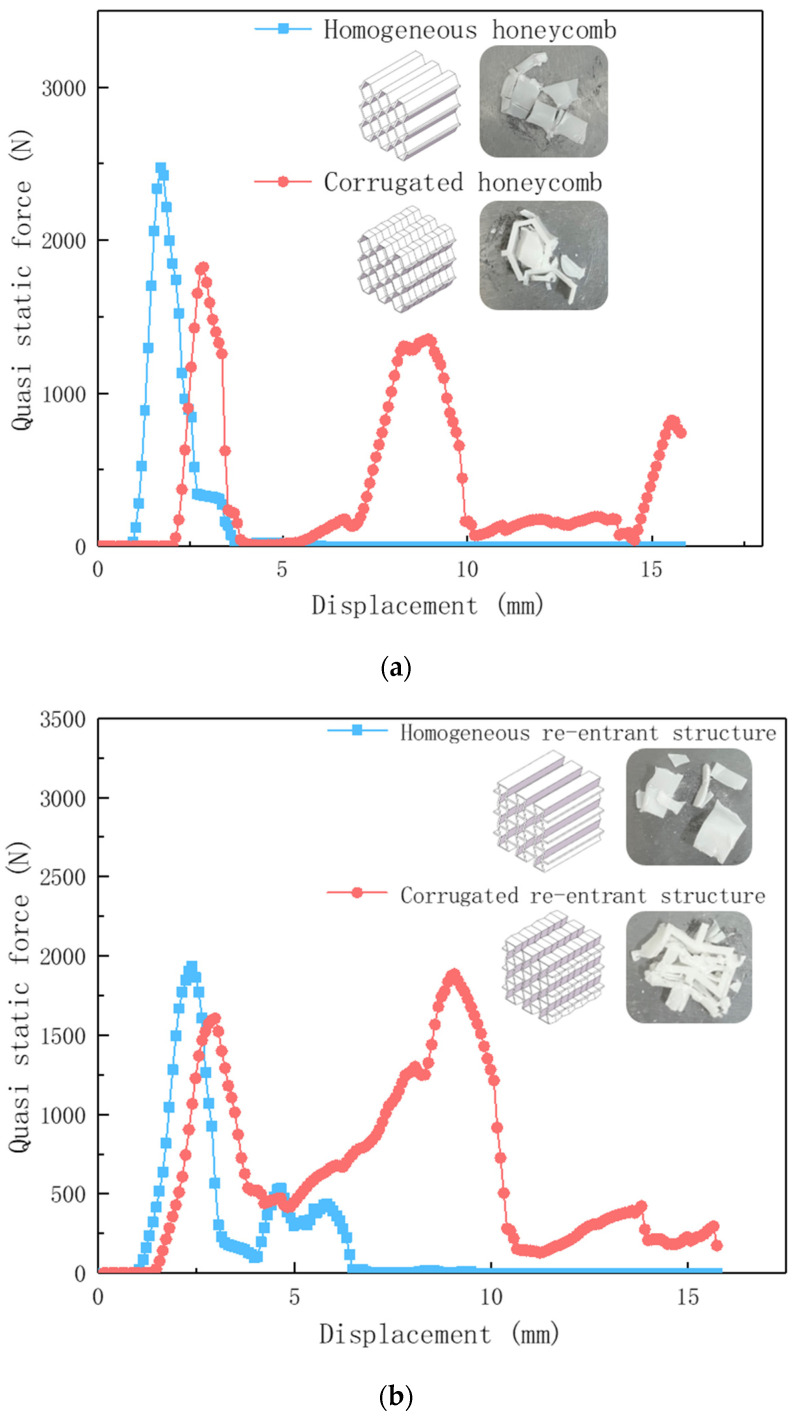

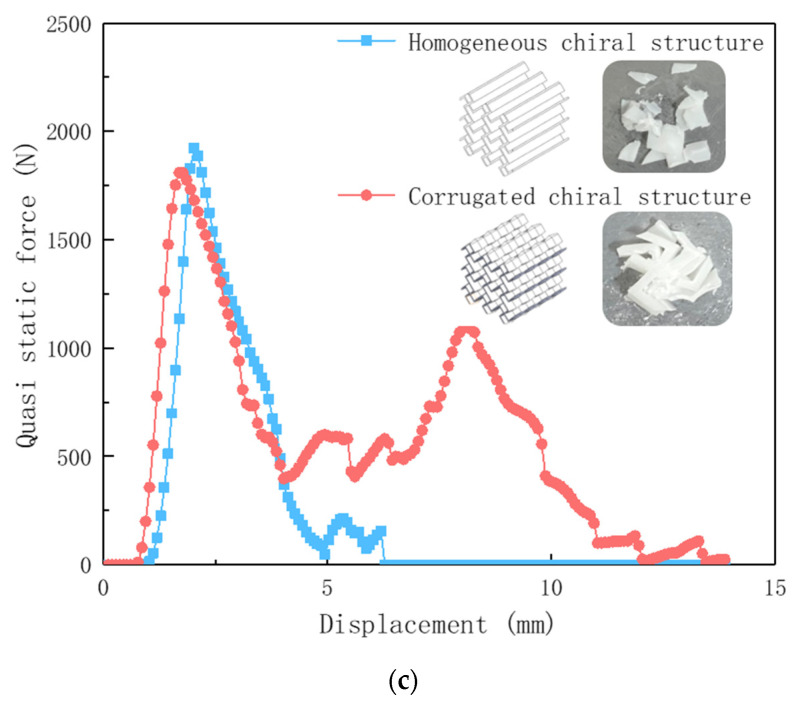
Bearing capacity change curve and failure morphology. (**a**) homogeneous and corrugated irregular hexagonal honeycomb comparison chart (**b**) homogeneous and corrugated re-entrant structure comparison chart (**c**) homogeneous and corrugated chiral structure comparison chart.

Figure 8a shows the bearing capacity change curve of the homogeneous irregular hexagonal structure and the corrugated irregular hexagonal structure at every moment. The homogeneous structures had higher load-bearing points. Then they quickly fell back and maintained a very low bearing level, and the structure basically lost its bearing capacity at this time. The overall pattern appeared to be a single narrow crest. After the first structural failure, the subsequent bearing capacity was insufficient and the structural integrity was almost completely lost. On the other hand, the maximum bearing capacity of the corrugated irregular hexagonal structure was lower than that of the homogeneous hexagonal structure, reaching 73.69%. This could be because the structure has a more complex distribution of forces. However, the corrugated structure had a second wave peak, and the highest bearing capacity of the second wave peak reached 73.76% of the first wave peak. The overall load-carrying capacity was higher, and complete structural damage did not occur after the initial impact. The structure still retained a certain level of load-carrying capacity even after the first impact. The damage morphology of the two samples was compared and analyzed. The uniform irregular hexagonal structure was completely damaged and the original morphology was lost, while the corrugated irregular hexagonal structure was seriously damaged. But, the sample morphology before damage was maintained to a certain extent, and the strength and supporting capacity were greatly weakened.

**Figure 9:**
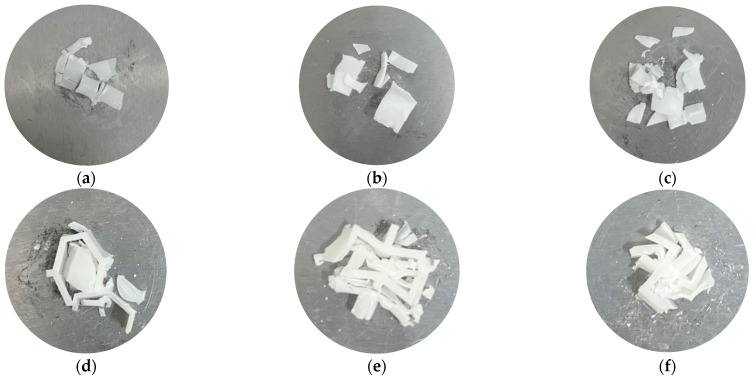
Sample failure morphology. (**a**) Homogeneous irregular hexagonal structure, (**b**) homogeneous reconstructed hexagonal structure, (**c**) homogeneous chiral structure, (**d**) corrugated irregular hexagonal structure, (**e**) corrugated reconstructed hexagonal structure, and (**f**) corrugated chiral structure.

Similarly, according to Figure 8b,c, it can be seen that the corrugated reconfigured hexagonal structure and the corrugated chiral structure have a “secondary peak” compared to the homogeneous reconfigured hexagonal structure and the homogeneous chiral structure. The sustained load-bearing capacity of the corrugated structure was greatly increased compared to the homogeneous structure, which reached the buckling limit and then the curve decreased rapidly. The structure still provided considerable load carrying capacity during the regularized collapse process. The analysis yields the percentage of load carrying capacity for both structures (see Table 3 below). The damage morphology of the corrugated reconfigured hexagonal structure (see Figure 9) was relatively intact, and the original morphology could be seen; the homogeneous structure was more damaged and fragmented, and the basic morphology was completely lost.

In addition, it could be found that the corrugated reconstruction hexagonal structure has a more specific phenomenon. The second wave peak was higher than the first wave peak, and the carrying capacity after local damage was better than that when the structure is stable. This might be because the reconstructed hexagonal structure had metamaterial properties [41,45]. After compression, the structure collapsed inward and densified, thereby increasing the load carrying capacity. The second wave crest of the wavy chiral structure was the lowest of the three samples. Based on the analysis of the damage morphology (as shown in Figure 9), it is speculated that the structure undergoes rotation during the compression process. The combined effect of multiple destructive forces accelerates the collapse of the structure. The observed damage morphology also indicates a spiral pattern around the intersection point, further supporting the rotational movement of the structure.

### 3.3. Energy Absorption Characteristics

**Figure 10:**
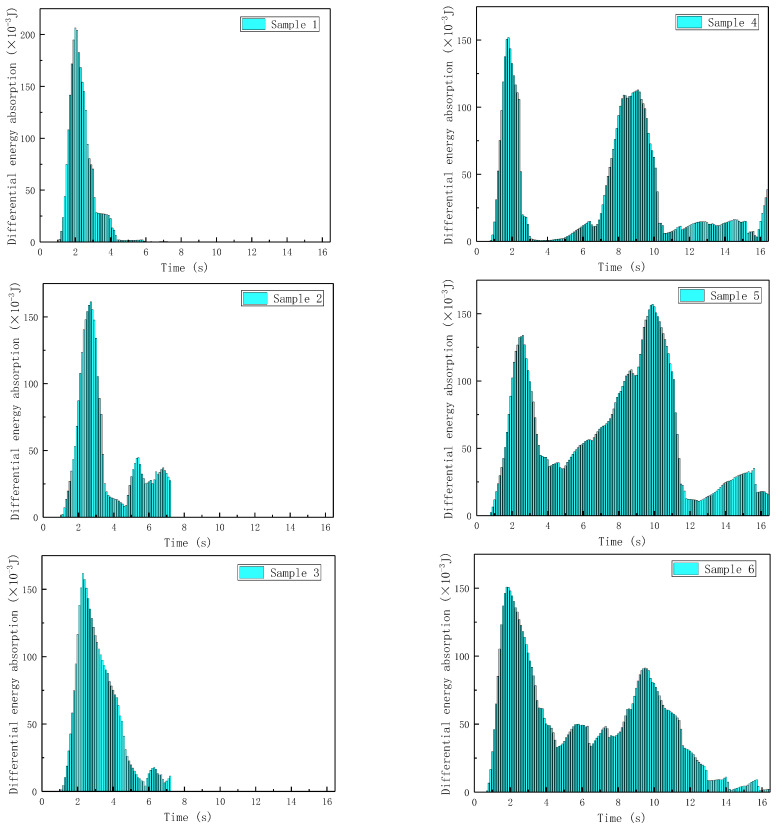
Time–absorption diagrams for 6 samples.

Each of the six samples corresponds to a (1) homogeneous irregular hexagonal structure, (2) homogeneous reconstructed hexagonal structure, (3) homogeneous chiral structure, (4) corrugated irregular hexagonal structure, (5) corrugated reconstructed hexagonal structure, (6) corrugated chiral structure. Based on Equations (7) and (8), the time–energy absorption differential maps of the six samples were plotted (Figure 10), and it could be seen that samples 1, 2, and 3 undergo rapid plastic collapse after elastic buckling, that there was almost no possibility of further load-bearing, and the energy absorption performance was greatly weakened until complete brittle damage occurred. Samples 4, 5, and 6 also experienced a reduction in load-bearing capacity after elastic buckling, but further elastic buckling was hindered by the influence of the corrugated structure during the plastic collapse process. Moreover, the buckling and collapse of the corrugated structure gradually extends from the weak point of the corrugated thickness to the maximum thickness, during which the honeycomb structure gradually densifies and undergoes relatively regular deformation; the transiently buckled structure was still able to provide a usable load-bearing capacity, and thus absorbed a considerable amount of energy. Due to the regularized deformation of the corrugated design, the lightweight structure obtained the load carrying capacity increase again after 6 s, the structure had a secondary energy absorption process, and the “secondary wave peak” appeared. The “secondary crest” delayed the brittle damage of the structure and at the same time gave the structure a continuous load-bearing and energy-absorbing capacity.

**Figure 11:**
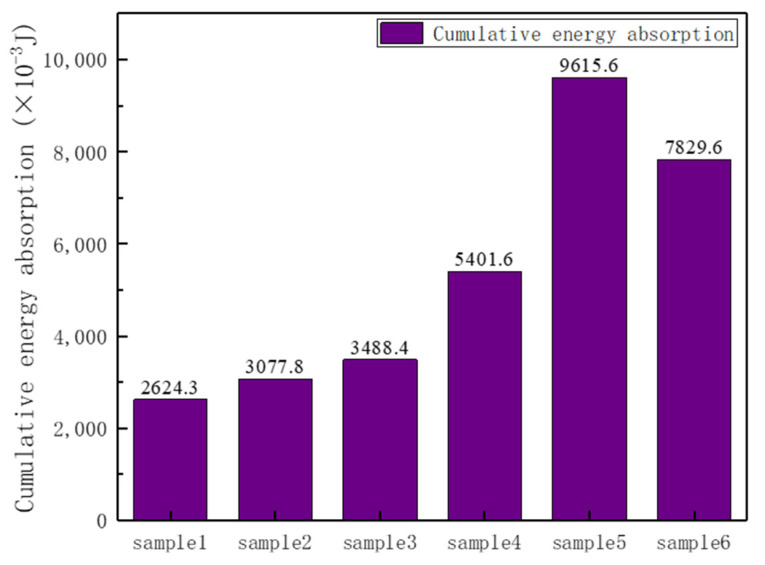
Cumulative energy absorption diagram.

The accumulation of time–absorbed energy was recorded for each instant, and the cumulative absorbed energy for the whole process time of the six samples was counted and compared (as shown in Figure 11). The cumulative energy absorption capacities of the corrugated bionic honeycomb lightweight structures were higher than those of the homogeneous structures of the same design, and the cumulative energy absorption capacities of samples 4, 5, and 6 were significantly higher than those of samples 1, 2, and 3 of the corresponding design. Z-directional corrugation was a very effective means of structural optimization. For the homogeneous structure samples 1, 2, and 3, both reconfiguration design and chiral design had better energy absorption than the hexagonal design, which can confirm the work of previous researchers.

**Figure 12:**
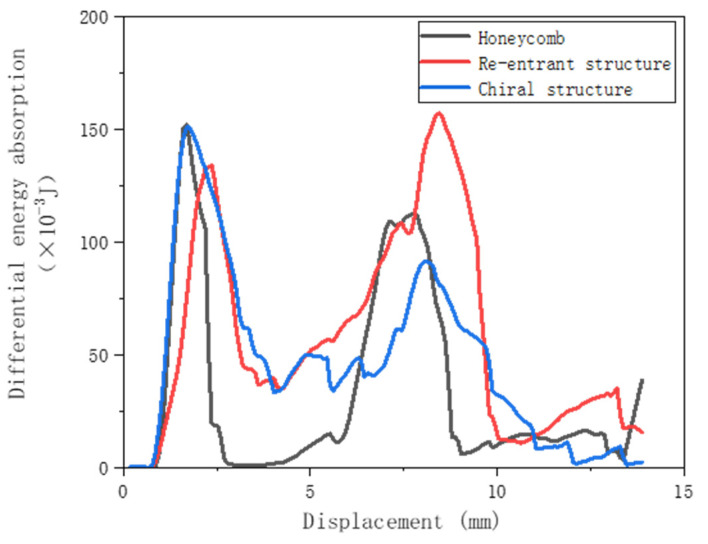
Comparison of energy absorption of three corrugated structures.

Further comparing the three designed corrugated reinforced lightweight structures (as shown in Figure 12), the differential energy absorption characteristics of the three corrugated structures were comparatively analyzed and the cumulative energy absorption diagrams were calculated. The cumulative energy absorption of the corrugated reconfiguration structure of the single-cell sample with h = 20 mm was enhanced by 78.01% compared with that of the corrugated hexagonal structure, and the cumulative energy absorption of the corrugated chiral structure was enhanced by 44.95% compared with that of the corrugated hexagonal structure. This indicated that the corrugated reconfiguration structure possesses the highest energy absorption effect among the three structures and was suitable to be designed preferentially, and that the honeycomb lightweight structure designed with dual reinforcement of corrugated and reconfiguration structures was an important direction to optimize the future development of aerospace honeycomb. At the same time, the results confirmed that the customized honeycomb and Z-direction corrugated design had greater advantages in the field of destructive impact and collision avoidance.

## 4. Conclusions

By simulating the initial deformation state and quasi-static compression test, the deformation force and failure starting point of the controlled deformation corrugated light honeycomb were studied. The energy absorption capacity and failure morphology of corrugated lightweight structures with different designs were analyzed and predicted. The performance difference of several samples was compared, which has reference significance for the custom design of the honeycomb core. The main conclusions of this study are as follows:

1. The corrugated structure lightweight honeycomb can realize the occurrence of deformation sensed in advance at the maximum stress–strain corrugation, which is conducive to the controllable deformation design of the overall structure. This in turn affects the time of occurrence of elastic buckling and delays the brittle damage process, so that the subsequent brittle damage of the overall structure is no longer “disorderly”;
2. After subjecting the corrugated lightweight structure to destructive impact, the presence of a second wave peak in the load-bearing capacity is observed. This characteristic is not exhibited by the homogeneous honeycomb structure. Furthermore, it can be inferred that the corrugated structure does not experience catastrophic damage as a result of the impact.;
3. The reconstructed corrugated lightweight structure may be due to the characteristics of the metamaterials. In particular, the peak carrying capacity of the second wave is 117.29% of the peak height of the first wave. The second bearing capacity of the chiral corrugated light structure is 60.7% of that of the first wave peak. However, the continuous load is strong, and the cumulative energy absorption is higher than that of the hexagonal structure;
4. The cumulative energy absorption of the corrugated reconstructed structure is 78.01% higher than that of the corrugated hexagonal structure. The cumulative energy absorption of the corrugated chiral structure is 44.95% higher than that of the corrugated hexagonal structure.

At present, the practical engineering application of this sample has not been widely used, and further test work is needed to explore the performance under more complex load conditions and multiple impact failure modes. This will be the next work plan.

## Figures and Tables

**Table 1:** Physical and mechanical properties of the light-curing resin.

Properties	Standard	Unit
Thermal deformation temperature	46	°C
Hardness	79	MPa
Tensile strength	47	MPa
Fracture strength	30–40	MPa
Tensile elongation ratio	3	%
Fracture elongation ratio	6–9	%
Elasticity modulus	2370–2650	MPa
Bending strength	69	MPa
Bending modulus	2178–2222	MPa
Impact strength	23–29	J/m^2^
Poisson ratio	0.41	-

**Table 2:** Meshing data of the models.

Model	Unit Total	Cell Size
homogeneous irregular hexagonal structure (model 1)	227,365	0.765551 mm
homogeneous reconstructed hexagonal structure (model 2)	385,907	0.6625 mm
homogeneous chiral structure (model 3)	363,523	0.644405 mm
corrugated irregular hexagonal structure (model 4)	792,305	0.482931 mm
corrugated reconstructed hexagonal structure (model 5)	750,016	0.53347 mm
corrugated chiral structure (model 6)	821,568	0.4954 mm

**Table 3:** Bearing capacity ratio of three typical structures.

	Proportion of First Bearing Capacity Peak of Corrugated/Homogeneous Structure/%	Corrugated Structure Second/First Bearing Capacity Peak Ratio/%
Irregular hexagonal structure	73.69	73.76
Reconstructed hexagonal structure	83.02	117.29
Chiral structure	94.10	60.70

## Data Availability

Data are contained within the article.
